# Phytotoxic Effects of Ethanolic Extracts of *Magonia pubescens* A.St.‐Hil. (Sapindaceae) on the Germination of *Emilia sonchifolia* (L.) DC. Seeds

**DOI:** 10.1002/cbdv.71644

**Published:** 2026-09-08

**Authors:** José Jonas Ferreira Viturino, Joice Barbosa do Nascimento, Debora Odilia Duarte Leite, Maria Eloyse de Melo Sousa, Jociany Carlos Caetano, Francisca Adriana Gomes dos Santos, Maria Arlene Pessoa da Silva, José Galberto Martins da Costa, Fabíola Fernandes Galvão Rodrigues, Raimundo Nonato Pereira Teixeira

**Affiliations:** ^1^ Graduate Program in Biological Diversity and Natural Resources Regional University Crato Ceará Brazil; ^2^ Natural Products Research Laboratory, Department of Biological Chemistry Regional University of Cariri Crato Ceará Brazil; ^3^ Graduate Program in Biological Chemistry, Department of Biological Chemistry Regional University of Cariri Crato Ceará Brazil; ^4^ Applied Botany Laboratory Regional University of Cariri Crato Ceará Brazil

**Keywords:** allelopathy, bioherbicide, phytotoxicity, plant extracts, secondary metabolism

## Abstract

*Emilia sonchifolia* (L.) DC. is a weed characterized by seed production and efficient wind dispersal. This study evaluated the allelopathic potential of extracts from the leaves, stem bark, and fruit pericarp (EECFMp) of *Magonia pubescens* on *E. sonchifolia* seed germination. HPLC‐DAD analysis identified caffeic acid in all extracts, while *p*‐coumaric and ferulic acids were detected in the stem bark and fruit pericarp extracts. The extracts exhibited concentration‐dependent activity. The leaf extract was the most phytotoxic, reducing germination to 33% at the highest concentration and outperforming glyphosate under the same conditions. Higher concentrations delayed germination (T50), reduced the germination speed index, and inhibited radicle and shoot growth. Lower concentrations stimulated germination, with the 50% stem bark extract achieving the highest germination rate (82%), indicating hormesis. These effects are likely associated with phenolic compounds, particularly caffeic acid. Overall, *M. pubescens* is a promising source of allelochemicals with potential application as a bioherbicide for weed management and as a seed germination biostimulant. Further investigations could elucidate the mechanisms of action and identify the specific compounds responsible for the phytotoxic and stimulatory effects.

## Graphical abstract

Ethanolic extracts from *Magonia pubescens* exhibit concentration‐dependent allelopathic activity against *Emilia sonchifolia*. Leaf extracts strongly inhibit seed germination and seedling growth, surpassing glyphosate under the tested conditions, whereas intermediate concentrations stimulate germination, indicating hormesis. Phenolic acids, particularly caffeic acid, are associated with the observed bioherbicidal and biostimulant potential.

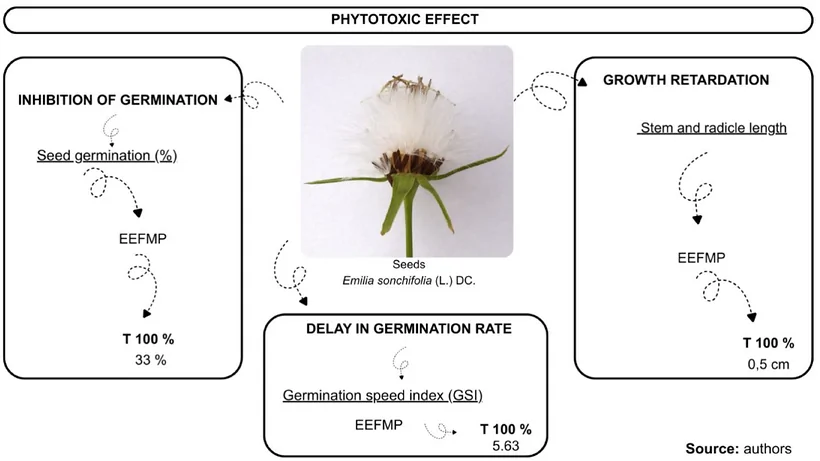

## 1 Introduction

Weeds are among the major challenges facing modern agriculture, causing substantial yield losses by competing with crops for essential resources and serving as natural reservoirs for pests. Moreover, the increasing occurrence of weed populations resistant to conventional herbicides has made their management progressively more difficult [1, 2].

*Emilia sonchifolia*, commonly known as “serralhinha,” is a moderately aggressive weed characterized by high seed production and efficient wind‐mediated seed dispersal. Native to tropical and subtropical regions of Asia, Africa, Polynesia, and the Americas, this species is widely distributed throughout Brazil, where it frequently occurs in agricultural fields, vacant lots, and urban environments [3].

The investigation of allelopathic interactions among plant species has become an important strategy in the search for novel herbicidal compounds. Several plant species, including *Eucalyptus urophylla* and *Mangifera* sp., have demonstrated inhibitory effects on seed germination and seedling development, reducing the establishment of target species and highlighting their potential as sustainable alternatives for weed management [4, 5]. Furthermore, allelochemicals generally present lower environmental risks than conventional herbicides because they are readily degraded in the environment and exhibit low persistence, thereby minimizing adverse effects on humans, animals, and ecosystems [6].

Despite advances in allelopathy research, studies correlating the chemical composition of plant extracts with their phytotoxic activity remain scarce, particularly those involving native Brazilian species with potential for bioherbicide development [7, 8]. Therefore, it was hypothesized that extracts of *M. pubescens* would exhibit concentration‐dependent allelopathic activity by modulating the germination and early seedling development of *E. sonchifolia* as a result of differences in the chemical composition among the evaluated plant organs.

Accordingly, the present study aimed to evaluate the allelopathic effects of ethanolic extracts obtained from the leaves, stem bark, and fruit peel of *M. pubescens* on the germination of *E. sonchifolia* seeds by assessing germination percentage, germination speed index, and radicle and shoot growth, with the objective of identifying the extract with the greatest potential for application in the development of natural bioherbicides.

## 2 Materials and Methods

### 2.1 Collection of Plant Material and Preparation of Extracts

Plant material was collected at Sítio Fundão State Park, located in Crato municipality, Ceará, Brazil (7°14′03.0″S, 39°25′56.2″W). A voucher specimen was deposited in the Herbário Caririense Dárdano de Andrade Lima (HCDAL) at the Regional University of Cariri (URCA) and identified by Dr. Maria Arlene Pessoa da Silva under voucher number 16 926.

The leaves, stem bark, and fruit peel of *M. pubescens* were separately processed to obtain the corresponding crude ethanolic extracts. For each plant material, 500 g of sample was subjected to static maceration in ethanol for 72 h at room temperature. Ethanol was selected as the extraction solvent to promote the recovery of chemical constituents with intermediate to high polarity, including phenolic compounds and other secondary metabolites potentially associated with the biological activities investigated.

Following maceration, the extracts were concentrated by removing the solvent under reduced pressure using a rotary evaporator. The resulting extracts were subsequently used in the analytical and/or biological assays described in the following sections.

Extraction yields were calculated as the ratio between the mass of the crude extract obtained after solvent removal and the initial mass of plant material and were expressed as percentages (w/w). Extraction yields of 4.42% were obtained for the ethanolic leaf extract of *M. pubescens* (EEFMp), 2.28% for the ethanolic stem bark extract (EECCMp), and 4.50% for the ethanolic fruit peel extract (EECFMp). Differences in extraction yields among the plant parts may be associated with variations in their chemical composition and the distribution of secondary metabolites across the different plant tissues.

### 2.2 Quantification of Phenolic Compounds and Flavonoids by HPLC‐DAD

HPLC analyses were performed using an Agilent 1260 system (Agilent Technologies, Germany) equipped with a diode array detector (DAD) and a C18 column (250 × 4.0 mm, 5 µm; Macherey‐Nagel, Germany). Separation was achieved using a gradient of ultrapure water (A) and methanol (60:40, v/v) (B), both acidified with 0.1% formic acid, at a flow rate of 0.5 mL/min. The gradient program was as follows: 15% B (0–15 min), 40% B (17 min), 30% B (30 min), and 15% B (38–45 min). The injection volume was 20 µL, and analyses were performed at room temperature in triplicate.

Samples were dissolved in HPLC‐grade methanol (30 mg/mL) and filtered through 0.22 µm membrane filters prior to injection. Phenolic compounds were identified by comparison of retention times and DAD spectra (190–400 nm) with those of reference standards. Quantification was performed using the external standard method based on analytical calibration curves prepared with the corresponding standards. Detection and quantification limits (LOD and LOQ) were calculated from three independent calibration curves using LOD = 3.3 *σ*/*S* and LOQ = 10 *σ*/*S*, where σ represents the standard deviation of the response and *S* the slope of the calibration curve.

### 2.3 Allelopathic Assay

#### 2.3.1 Preparation of Concentrations

For the allelopathic assays, the extracts (EEFMp, EECCMp, and EECFMp) were prepared according to Lazazzara et al. [9], with modifications. Crude extract (1 g) was dissolved in 100 mL of distilled water to obtain the 100% treatment (10 mg/mL). From this stock solution, the following dilutions were prepared to obtain the 75%, 50%, and 25% treatments:

$$\begin{array}{ccc} & & 75\%\left(7.5mg/mL\right)-7.5ml\;of\;stock\;solution\\ & & +2.5mL\;distilled\;water \end{array}$$

$$50\%\left(\frac{5mg}{mL}\right)-5mL\;of\;stock\;solution+5mL\;distilled\;water$$

$$\begin{array}{ccc} & & 25\%\left(\frac{2.5mg}{mL}\right)-2.5mL\;of\;stock\;solution\\ & & +7.5mL\;distilled\;water \end{array}$$

#### 2.3.2 Performing the Test

For the seed germination bioassays, the method described by Alves et al. [10] and Bonato et al. [11] was used, with modifications. For the ethanolic extracts of *M. pubescens*, seeds of *E. sonchifolia* (L.) DC. were used as the recipient species. The seeds were collected on the campus of the Regional University of Cariri (URCA) (7°14′19″ S – 39°24′55″ W), stored in a cool, dry environment, and subsequently surface‐sterilized in a 5% sodium hypochlorite (NaClO) solution, followed by washing in running water prior to setting up the experiment.

Bioassays were conducted in quintuplicate with 20 seeds per treatment, arranged in a completely randomized design. Seeds were placed equidistantly on previously sterilized Petri dishes containing two sheets of Germitest paper (purchased from a gardening store) for germination. Each plate received 5 mL of the respective extract for each treatment, and distilled water was added as needed to maintain appropriate conditions for germination. The control group consisted of distilled water only, and a positive control was performed using a commercial glyphosate‐based herbicide at the same concentrations as the extracts (100%, 75%, 50%, and 25%).

Plates were incubated in biochemical oxygen demand (BOD) growth chambers at a constant temperature of 25°C with a 12‐h photoperiod (light/dark). The number of germinated seeds was recorded daily for 8 days, with seeds considered germinated when the radicle protruded at least 2 mm. The following parameters were evaluated: germination percentage (G), shoot and radicle length, and germination speed index (GSI). Germination percentage was calculated using the formula:

G=N/A×100

where *G* = germinability (%), *N* = number of germinated seeds, and *A* = total number of seeds sown [12]. The germination speed index (GSI) was calculated according to Maguire [13] using the formula:

GSI=G1/N1+G2/N2+..Gn/Nn

where *G*1, *G*2,…, *Gn* correspond to the number of seeds germinated on each day after sowing [14].

Mean Germination Time (MGT) was calculated according to the equation proposed by Labouriau [15].

TMG=ni.ti/ni

where MGT = mean germination time (days); ni = number of seeds germinated within a time interval; and ti = germination time interval.

### 2.4 Statistical Analysis

One‐way analysis of variance (ANOVA) was used for statistical analysis, and means were compared using Tukey's test at a 5% probability level (*p* < 0.05). The data were subjected to normality tests (D'Agostino and Pearson, Shapiro‐Wilk, and Kolmogorov–Smirnov). Data that did not meet the normality assumptions were analyzed using the Kruskal–Wallis test, followed by Dunn's post‐hoc test (*p* < 0.0001 relative to the negative control). Values were expressed as median and interquartile range (*n* = 25).

Statistical analyses were performed using Assistat (version 7.7), BioEstat (version 5.0), and GraphPad Prism (version 8.0.1) software.

## 3 Results and Discussion

### 3.1 Analysis Using HPLC‐DAD

**FIGURE 1:**
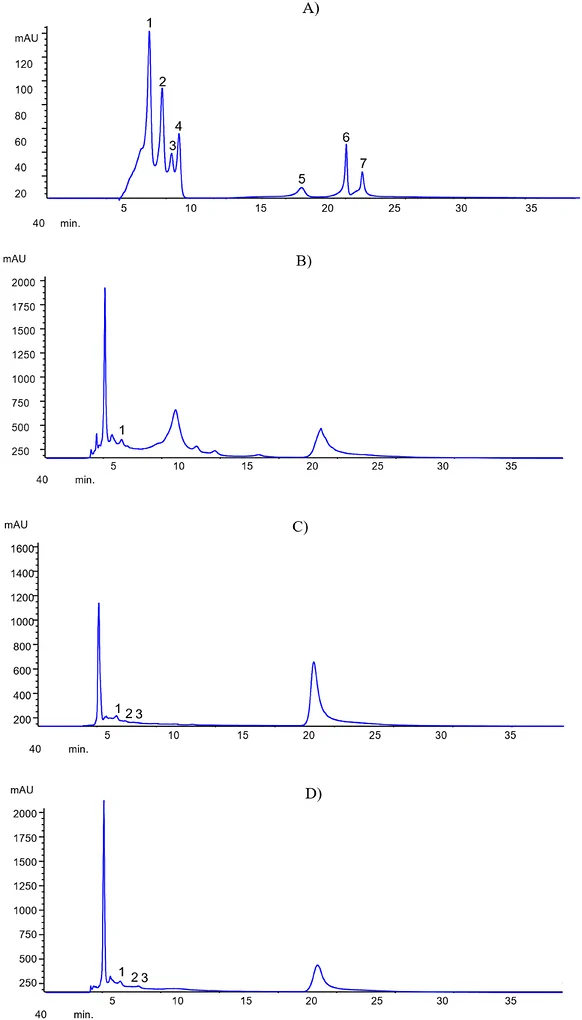
(A) HPLC profile of phenolic acid and flavonoid standards. Caffeic acid (tR = 6.6 min, peak 1), *p*‐coumaric acid (tR = 7.3 min, peak 2), ferulic acid (tR = 7.8 min, peak 3), cinnamic acid (tR = 8.3 min, peak 4), naringenin (tR = 15.2 min, peak 5), pinocembrin (tR = 19.1 min, peak 6) and apigenin (tR = 24.1 min, peak 7). Calibration curve for caffeic acid: *y* = 331.13*x* − 94.735 (*r* ^2^ = 0.9999); *p*‐coumaric acid: *y* = 449.98*x* − 72.528 (*r* ^2^ = 0.9971); ferulic acid: *y* = 185.3*x* − 675.79 (*r* ^2^ = 0.9887); cinnamic acid: *y* = 236.74*x* − 188.67 (*r* ^2^ = 0.9991); naringenin: *y* = 151.64*x* + 8.5796 (*r* ^2^ = 0.9994); pinocembrin: *y* = 177.66*x* − 390.72 (*r* ^2^ = 0.9955) and apigenin: *y* = 164.9*x* − 688.69 (*r* ^2^ = 0.9963). (B) HPLC profile of EEFMp, (C) HPLC profile of EECCMp; and (D) HPLC profile of EECFMp.

The results of the chromatographic analyses are presented as chromatograms for each extract (Figure 1). Figure 1A shows the chromatographic profile of phenolic acid standards (*p*‐coumaric acid, ferulic acid, and cinnamic acid) and flavonoids (naringenin, pinocembrin, and apigenin). Caffeic acid (peak 1) was identified in all three analyzed samples, with concentrations of 0.0167 mg/g in EEFMp (Figure 1B), 0.1593 mg/g in EECCMp (Figure 1C), and 0.1537 mg/g in EECFMp (Figure 1D). Other compounds, such as *p*‐coumaric acid (peak 2) and ferulic acid (peak 3), were detected only in EEFMp and EECFMp extracts, with concentrations reported in Table 1.

**TABLE 1:** Quantification by HPLC/DAD of phenolic acids and flavonoids.

Compound	LD (mg/mL)	LQ (mg/mL)	EEFMp		EECCMp		EECFMp	
			(mg/g)	(%)	(mg/g)	(%)	(mg/g)	(%)
Caffeic acid	0.0001	0.0003	0.1667 ± 0.0676	0.0167	0.1593 ± 0.0012	0.0159	0.1537 ± 0.0072	0.0154
*p‐*Coumaric acid	0.0007	0.0025	nd	nd	0.0106 ± 0.0000	0.0011	0.0069 ± 0.0000	0.0007
Ferulic acid	0.0048	0.0162	nd	nd	0.1406 ± 0.0000	0.0141	0.1917 ± 0.0055	0.0192
Cinnamic acid	0.0009	0.0032	nd	nd	nd	nd	nd	nd
Naringenina	0.0011	0.0036	nd	nd	nd	nd	nd	nd
Pinocembrine	0.0030	0.0102	nd	nd	nd	nd	nd	nd
Apigenin	0.0027	0.0092	nd	nd	nd	nd	nd	nd
Total			0.1667	0.0167	0.3105	0.0311	0.3523	0.0352

*Note*: Results are expressed as mean (mg/g of sample) ± SD (*n* = 3).

Abbreviation: nd, not detected.

Caffeic acid, *p*‐coumaric acid, and ferulic acid stand out as potent antioxidant agents with antimicrobial, insecticidal, and allelopathic activity, as well as roles in anti‐inflammatory, neuroprotective, antiviral, and anticarcinogenic processes, with applications across multiple economic sectors including agriculture, food, cosmetics, and pharmaceuticals [16, 17, 18].

Although chemical studies on *M. pubescens* are still limited, they reveal the presence of important metabolite classes, including tannins, saponins, flavonoids, alkaloids, and triterpenes in the leaves [19]; fatty acids in the seeds [20]; and catechin‐type tannins in the stem bark [21]. In the study by Morais et al., [22] chemical analysis of extracts and fractions from *M. pubescens* branches using UHPLC revealed numerous compounds, including quercetin and afzelin (flavonoids), aleuritine and glycyrrhetinic acid (triterpenes), fraxetin and scopoletin (coumarins), among other compounds detected in the analysis.

### 3.2 Seed Germination

**FIGURE 2:**
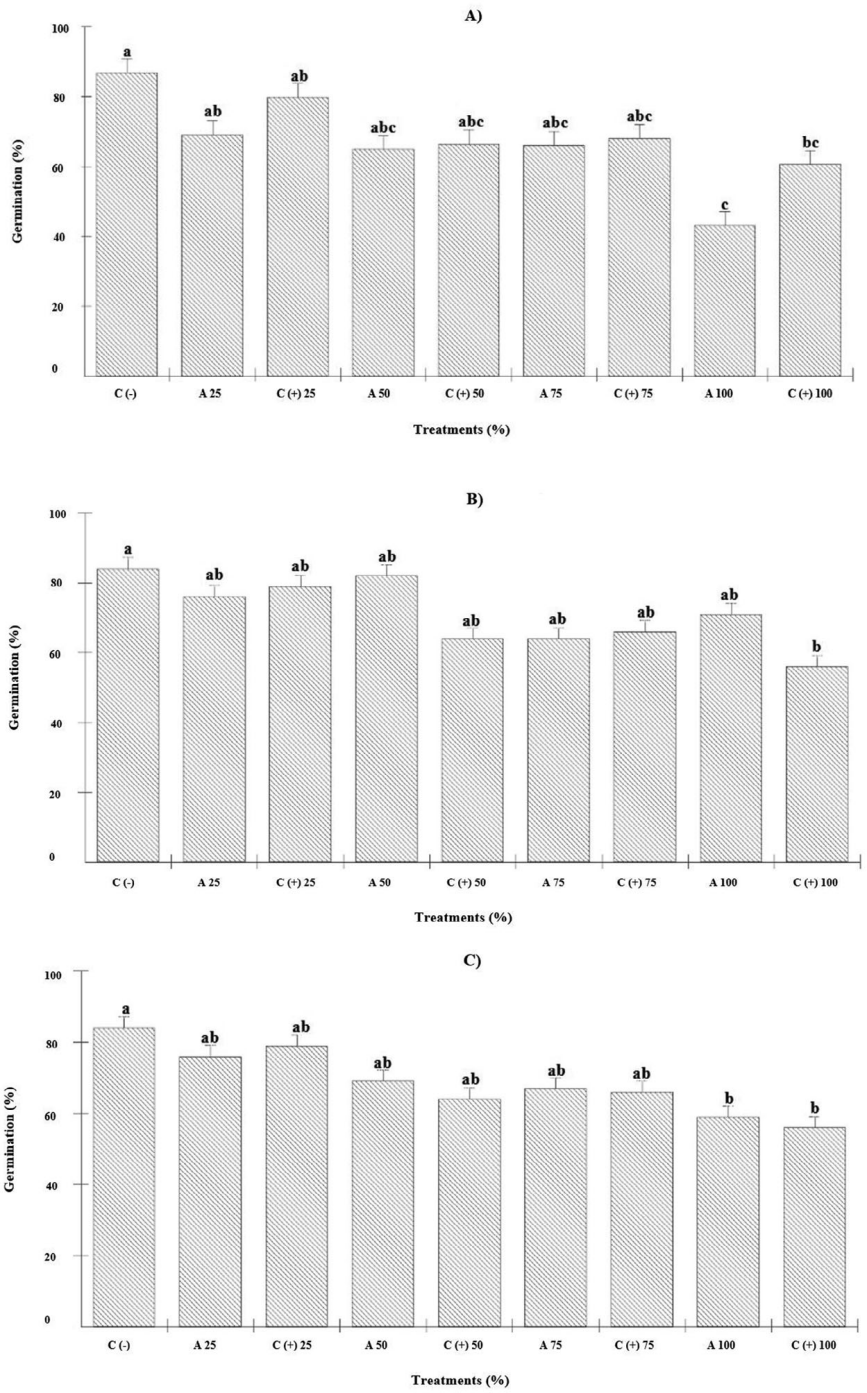
Percentage of seed germination by treatment (negative control, water; positive control, glyphosate‐based herbicide). In (A), the values for seed germination of the leaf extract (EEFMp) are described; in (B), the values for the stem bark extract (EECCMp) are described; and in (C), the values for the fruit peel extract (EECFMp) are described. The values represent the mean ± standard error (SD) for trials/group. The bars indicate the standard error. Values followed by different letters are significantly different according to the one‐way analysis of variance (ANOVA) followed by Tukey's test (*p* ≤ 0.05).

The results demonstrate that ethanolic extracts of *Magonia pubescens* exert distinct effects on the germination of *E. sonchifolia* seeds depending on extract concentration (Figure 2). In Figure 2A, corresponding to the EEFMp extract, an approximate 50% reduction in seed germination was observed compared with the negative control (water), and a 40% reduction compared with the positive control (glyphosate) at the 100% extract treatment.

A similar effect was observed for the other extracts, EECCMp (Figure 2B) and EECFMp (Figure 2C), though to a lesser extent compared with the negative control. When compared with the positive control, the extracts were less effective. At lower concentrations (Table 2), the extracts exhibited positive effects on seed germination, increasing germination rates above those of the negative control, with the EECCMp extract at 50% being the most effective (82%). Table 2 also shows that, when compared with the positive control, the seeds of the receptor species exhibited resistance to the glyphosate‐based herbicide, indicating that at low concentrations, the herbicide did not achieve the expected effects on weeds.

**TABLE 2:** Seed germination of *E. sonchifolia* under the effect of *M. pubescens* extracts.

	Seed germination (%)			
Treatment	EEFMp	EECCMp	EECFMp	Control (+)
**25%**	67	76	76	79
**50%**	61	82	69	64
**75%**	63	64	67	66
**100%**	33	71	59	56
Control (‐) 70%				

*Note*: Control (‐), H_2_O. Results were expressed as a percentage (%). Control (+), glyphosate herbicide; EEFMp, ethanolic extract of *M. pubescens* leaves; EECCMp, ethanolic extract of *M. pubescens* stem bark; EECFMp, ethanolic extract of *M. pubescens* fruit peel.

The results indicate that even at the maximum product concentration (10 mg/mL), the herbicide did not produce significant inhibitory effects. In contrast, under the same conditions, the EEFMp extract demonstrated negative allelopathic effects on *E. sonchifolia* seed germination, performing better than glyphosate under the same treatment conditions, revealing its potential for weed control.

Although studies on the allelopathic effects of *M. pubescens* are still limited, these results corroborate previous findings. Alves et al. [10] evaluated the effects of hydrogel derived from *M. pubescens* seeds on *Cucumis sativus* (cucumber) seed germination and found that germination percentage decreased with increasing hydrogel concentration, with the most pronounced effect at 100% hydrogel (only 63% germination). Conversely, lower hydrogel concentrations promoted higher germination rates, with 50% hydrogel achieving 93% germinated seeds.

These findings indicate the potential of the species for use as a natural biofertilizer at lower concentrations, increasing germination rates for agricultural crops with low germination or for native seeds in reforestation areas. Another relevant agricultural application is in weed control, where higher extract concentrations may act as a source of allelochemicals capable of inhibiting or delaying seed germination.

In another study, Bonato et al. [11] evaluated variability in *Sorghum bicolor* (sorghum) seed germination under high hydrogel concentrations from *M. pubescens* seeds and observed that varying extract concentrations resulted in different effects on seed germination. According to the authors, this may be associated with a pharmacokinetic phenomenon known as hormesis, in which the compound exhibits different biological effects depending on its concentration.

Based on the chemical composition described in this study and supported by literature, the allelopathic effects of the identified compounds can be highlighted. Li et al. [23] evaluated the inhibition of *Microcystis aeruginosa*, a freshwater cyanobacterium, by six phenolic acids derived from plant secondary metabolites, including caffeic acid, cinnamic acid, and *p*‐coumaric acid. Among these, caffeic acid demonstrated the most efficient growth inhibition, with an EC50 of 5.8 mg/L after 96 h of exposure.

#### 3.2.1 Mean Germination Time (MGT)

As shown in Table 3, the mean germination time (MGT) increased with increasing extract concentration, indicating a delay in the germination process of *E. sonchifolia* seeds. Among the evaluated extracts, the ethanolic leaf extract (EEFMp) exhibited the strongest effect, reaching 6.88 ± 0.89 days at the 100% concentration, whereas the stem bark (EECCMp) and fruit peel (EECFMp) extracts produced less pronounced increases in MGT. Compared with the positive control, EEFMp delayed germination more markedly at the 25%, 50%, and 100% concentrations, corroborating the results obtained for germination percentage and the germination speed index (GSI). These findings demonstrate that the leaf extract possesses the greatest allelopathic potential, delaying the initial establishment of seedlings.

**TABLE 3:** Mean germination time (MGT) of seeds treated with *M. pubescens* extracts.

	MGT (day)			
Treatment	EEFMp	EECCMp	EECFMp	Control (+)
**25%**	6.39 ± 0.36^a^ ^b^	5.50 ± 0.41^a^	5.44 ± 0.41^a^	4.84 ± 0.40^b^
**50%**	6.54 ± 0.29^a^ ^b^	6.08 ± 0.46^a^	5.66 ± 0.30^a^	5.18 ± 0.69^a^ ^b^
**75%**	5.65 ± 0.35^a^ ^b^	6.14 ± 0.43^a^	5.96 ± 0.38^a^	5.70 ± 0.39^a^ ^b^
**100%**	6.88 ± 0.89^b^	6.05 ± 0.19^a^	6.48 ± 0.50^a^	6.35 ± 0.65^a^
Control (‐) 5,49 ± 1,25^a^ ^b^				

*Note*: Means followed by the same letter in the column do not differ from each other according to Tukey's test (*p* < 0.05). Only the 100% and 25% treatments differed significantly from each other (*p* = 0.0368).

The increase in MGT associated with the reduction in GSI indicates that the extracts altered the germination dynamics of *E. sonchifolia* seeds. Although the evaluated parameters do not allow the precise site of action of the allelochemicals to be determined, the results suggest that the extracts acted predominantly during the metabolic phases following seed imbibition, delaying radicle protrusion. According to Kostina–Bednarz, Płonka, and Barchanska [8], allelochemicals, particularly phenolic compounds, may interfere with reserve mobilization, the activity of hydrolytic enzymes, cellular respiration, hormonal balance, and redox homeostasis, thereby impairing germination rate and the initial establishment of seedlings.

The greater activity observed for EEFMp may be associated with differences in the chemical composition among the plant organs of *M. pubescens*. Although caffeic acid was identified in all extracts, whereas *p*‐coumaric and ferulic acids were detected only in the stem bark and fruit peel extracts, the superior activity of the leaf extract suggests that other secondary metabolites or synergistic interactions among its constituents may have enhanced its phytotoxic activity. According to Kostina–Bednarz, Płonka, and Barchanska [8], allelopathic activity depends not only on the chemical nature of the allelochemicals but also on their concentration, the interactions among metabolites within the extract, and the sensitivity of the target species, all of which determine the magnitude of the phytotoxic response.

Overall, these findings reinforce the potential of the ethanolic leaf extract of *M. pubescens* as a promising source of bioactive compounds for the development of natural bioherbicides. However, further physiological, biochemical, and molecular studies are required to elucidate the mechanisms of action of the allelochemicals and to identify the metabolites responsible for the observed effects, as recommended by recent reviews on allelopathy and bioherbicide development.

#### 3.2.2 Germination Time of 50% of Seeds (T50)

**FIGURE 3:**
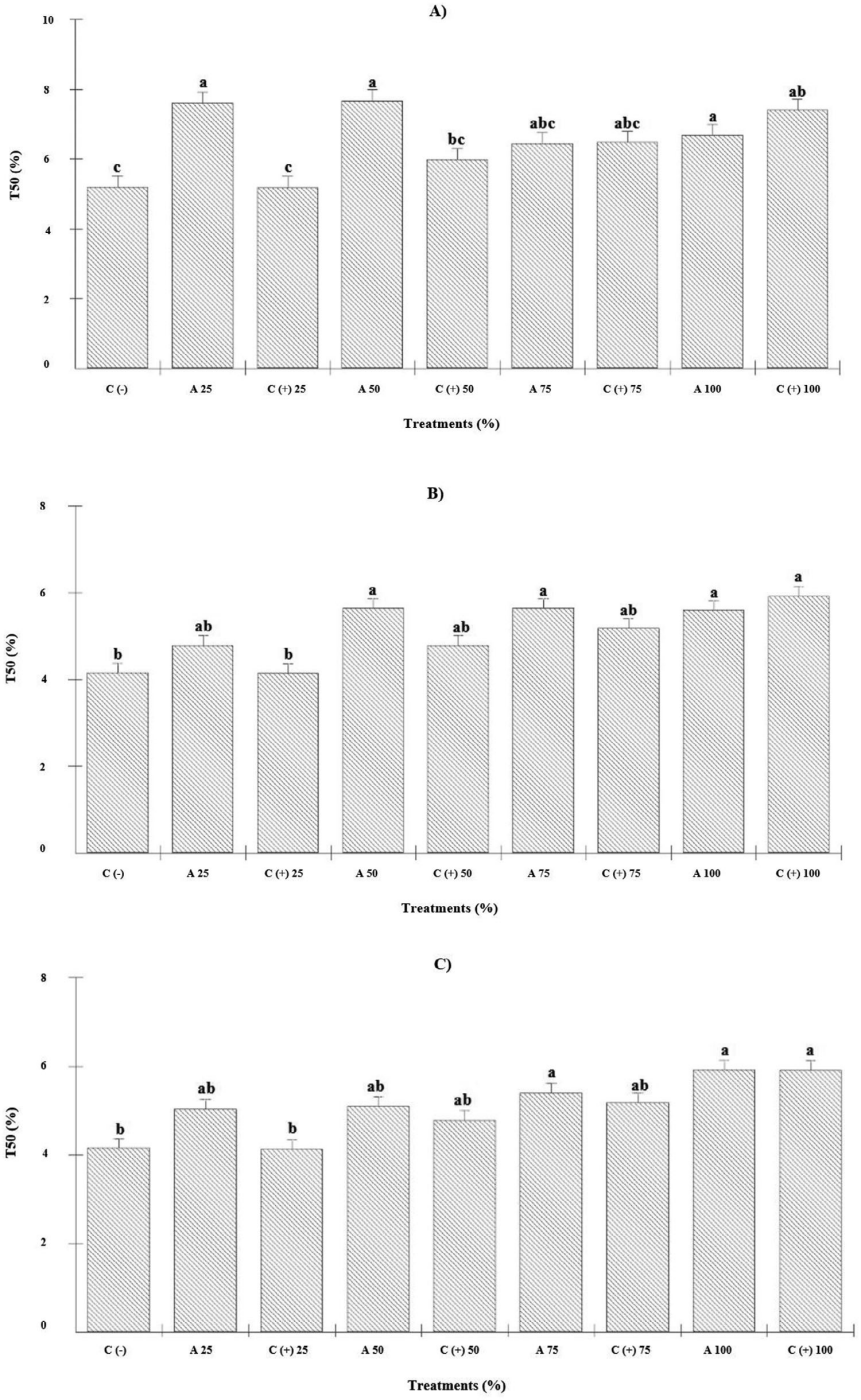
Germination time of 50% of seeds (T50). In (A), the germination values of the leaf extract (EEFMp) are described; in (B), the values referring to the stem bark extract (EECCMp); and in (C), the values referring to the fruit peel extract (EECFMp). The values represent the mean ± standard error (SD) for trials/group. The bars indicate the standard error. Values followed by different letters are significantly different according to the one‐way analysis of variance (ANOVA) followed by Tukey's test (*p* ≤ 0.05).

The evaluation of the time required for 50% of *E. sonchifolia* seeds to germinate (T50) demonstrated significant effects influenced by the different treatments tested. The negative control presented one of the lowest T50 values, indicating a faster germination process, which differed statistically from treatments with higher extract concentrations. For the EEFMp extract (Figure 3A), the 25% and 50% treatments exhibited the highest T50 values, indicating a significant effect on germination time. Even at intermediate concentrations, the extract was able to delay the germination process, suggesting an inhibitory effect on germination speed.

For the 75% treatment, an intermediate behavior was observed, with no clear statistical difference compared with the positive control. In contrast, the 100% treatment showed elevated T50 values, similar to those observed at 25% and 50%, indicating that higher extract concentrations prolong the time required for germination.

For the EECCMp extract (Figure 3B), the 25% and 50% concentrations showed intermediate T50 values and did not differ statistically from the positive control, suggesting that these concentrations exerted a limited effect on germination speed. This behavior indicates that at lower concentrations, the extract does not markedly impair the initial germination process. Conversely, the 75% and 100% treatments, as well as their respective positive controls, exhibited the highest T50 values, suggesting an inhibitory effect on germination speed.

Regarding the EECFMp extract (Figure 3C), the 25% and 50% treatments presented intermediate T50 values and did not differ statistically from their corresponding controls, suggesting that at these concentrations the extract did not substantially delay seed germination. In contrast, the 75% treatment resulted in a significant increase in T50, evidencing delayed germination at higher concentrations. The highest T50 values were observed in the 100% extract treatment and in the 100% positive control.

Overall, these results demonstrate a significant delay in the germination of *E. sonchifolia* seeds, suggesting an inhibitory effect associated with increasing extract concentration. This pattern indicates a dose‐dependent response, in which higher concentrations promote delayed germination. The increase in T50 at elevated concentrations may be related to the presence of bioactive compounds with allelopathic or phytotoxic potential, capable of interfering with essential physiological processes involved in germination, such as water uptake, enzymatic activity, and early embryonic growth. Thus, the findings reinforce the hypothesis that the extract exerts a negative effect on germination speed at higher concentrations.

### 3.3 Germination Speed Index (GSI)

The analysis revealed effects similar to those observed for germination, with a reduction in the germination speed index (GSI) at higher extract concentrations compared with the negative control (Figure 3, Table 4). Thus, increasing extract concentrations resulted in greater interference of allelochemicals on the germination rate of *E. sonchifolia* seeds, as previously estimated by the T50 values of the extracts [8].

**TABLE 4:** Germination speed index (GSI) of *E. sonchifolia* seeds under the effect of *M. pubescens* extracts.

	Germination speed index (GSI)			
Treatment	EEFMp	EECCMp	EECFMp	Control (+)
**25%**	7.04	9.22	9.30	10.59
**50%**	7.22	8.96	8.98	8.56
**75%**	8.58	7.35	8.36	8.61
**100%**	5.63	8.00	7.21	6.65

*Note*: Control (+), glyphosate herbicide; (EEFMp), ethanolic extract of *M. pubescens* leaves; (EECCMp), ethanolic extract of *M. pubescens* stem bark; (EECFMp), ethanolic extract of *M. pubescens* fruit peel.

**FIGURE 4:**
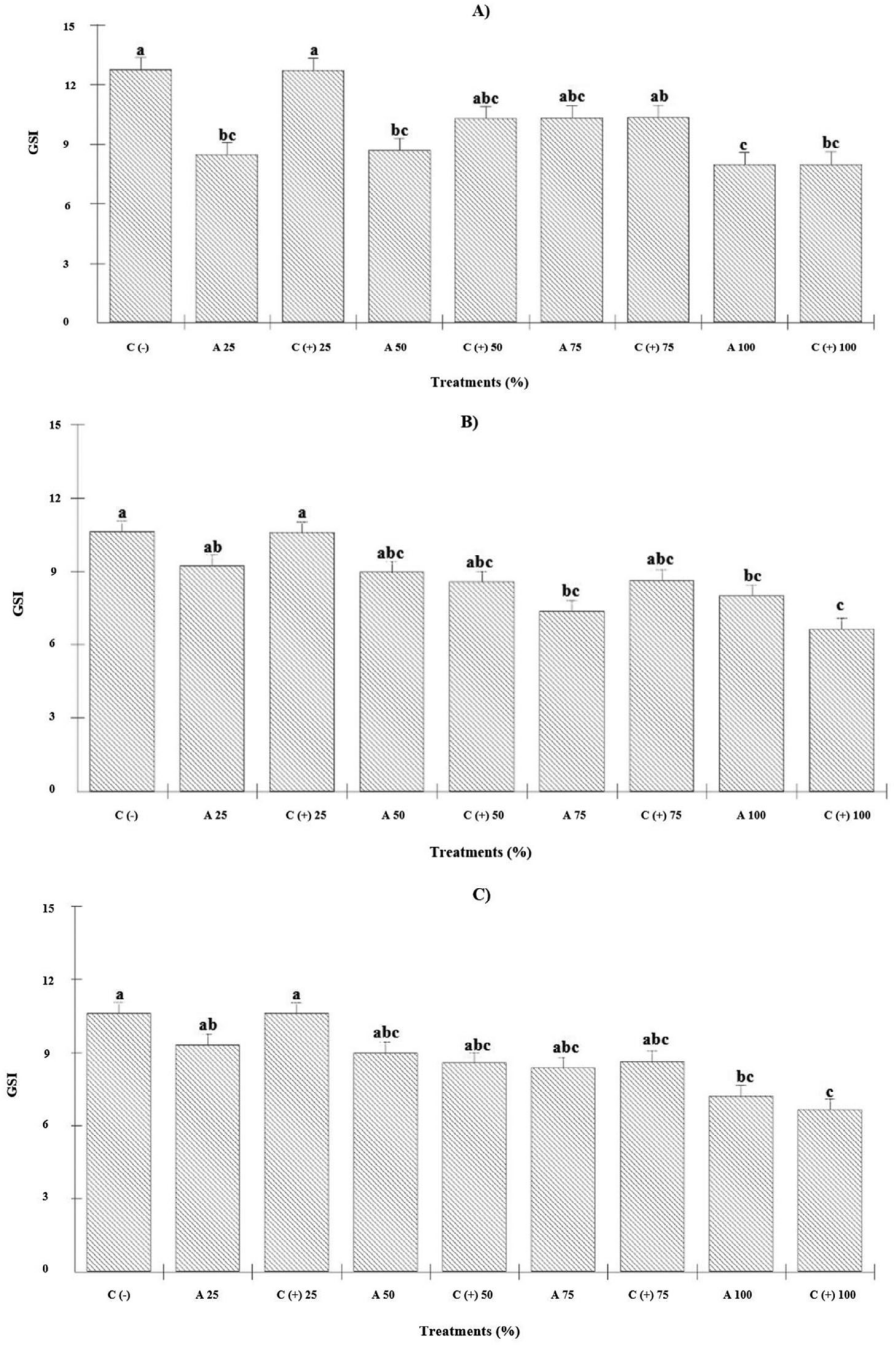
Germination speed index (GSI). C (‐), negative control: water; C (+), positive control: glyphosate‐based herbicide; A25, A50, A75, and A100: treatments. In (A), the GSI values of the leaf extract (EEFMp) are described; in (B), the GSI values of the stem bark extract (EECCMp); and in (C), the GSI of the fruit peel extract (EECFMp). The values represent the mean ± standard error (SD) for trials/group. The bars indicate the standard error. Values followed by different letters are significantly different according to the one‐way analysis of variance (ANOVA) followed by Tukey's test (*p* ≤ 0.05).

When comparing the data from the positive control with the extracts, the EEFMp extract (Figure 4A) showed variation at the 25% and 50% treatments, where the extract was more effective, with GSI values of 7.04 and 7.22, respectively, indicating delayed seed germination. The greatest effect was observed at the 100% treatment, with a GSI of 5.63. At the 75% treatment, both showed similar GSI values.

The EECCMp (Figure 4B) and EECFMp (Figure 4C) extracts were more effective at the 25% and 75% treatments compared with the positive control. The GSI data presented in Table 3 suggest that although *M. pubescens* extracts exhibit concentration‐dependent effects, even the lowest treatments interfered negatively with the germination of *E. sonchifolia* seeds when compared with the negative control. Furthermore, even in the presence of a glyphosate‐based herbicide, no significant changes were observed in its effects on seed germination, particularly at the 25% treatment, where the GSI was similar to the water control [7].

When comparing the analyzed extracts, EEFMp was the most effective, significantly delaying the germination rate of *E. sonchifolia* seeds at the 100% treatment, consistent with the results described for the germination index. These findings indicate that this extract may be a promising candidate for weed control. Among the factors potentially associated with the greater effects of EEFMp on seed germination is the presence of phenolic compounds identified in the chemical analysis [17, 24].

### 3.4 Stem and Radicle Length

Data referring to shoot and radicle length were assessed for normality, and due to the absence of normal distribution, the Kruskal–Wallis test was applied, followed by Dunn's post hoc test comparing each treatment with the negative control, adopting a significance level of α = 0.05. Five experimental groups were analyzed, with 25 replicates per group (*n* = 125). The Kruskal–Wallis test indicated a statistically significant difference among groups, demonstrating that at least one treatment significantly affected shoot and radicle length.

High variability was observed in the negative control data, reflected by the wide dispersion of shoot and radicle length values. This variation may be attributed to biological heterogeneity among samples, environmental fluctuations during the experiment, or other uncontrolled factors. Despite the high variability, it did not compromise the statistical analyses, and significant differences among the tested groups were detected.

**FIGURE 5:**
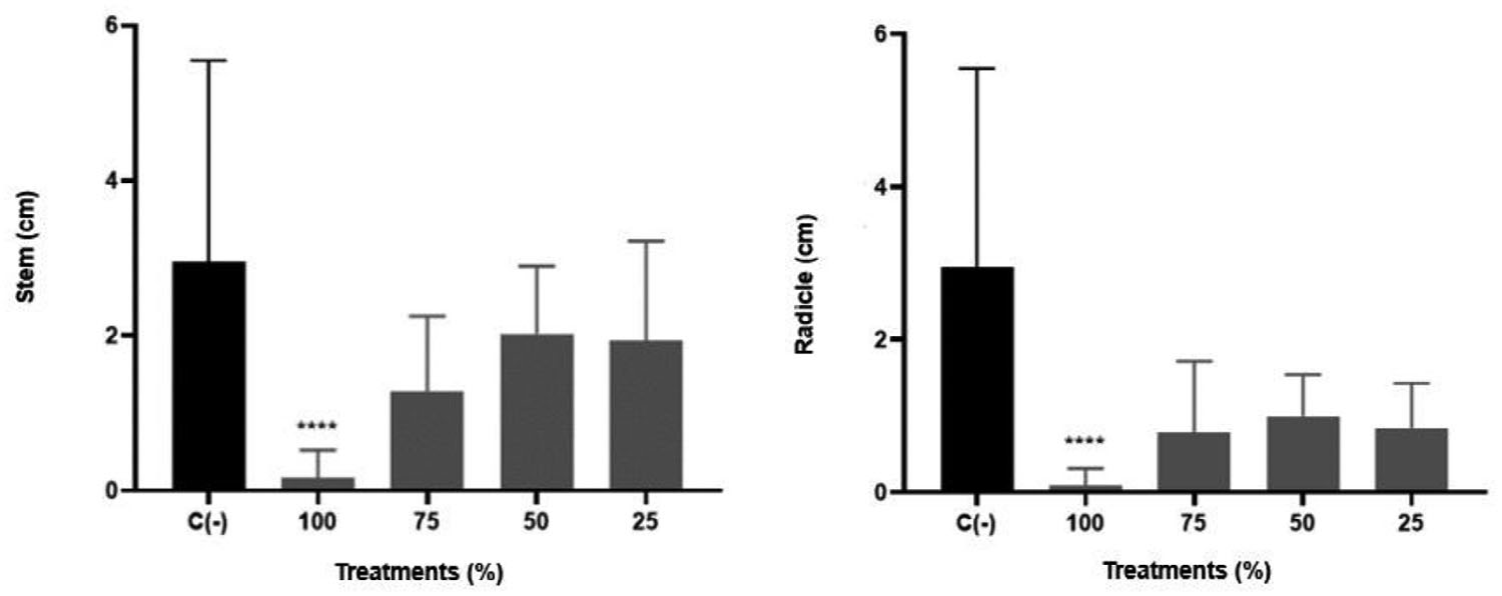
Stem and radicle length (cm) of the EEFMp extract. Values expressed as median and interquartile range (*n* = 25). Differences between groups were assessed using the Kruskal–Wallis test, followed by Dunn's post‐hoc test. ****p < 0.0001 compared to the negative control.

Graphical analysis of the results obtained for the EEFMp extract (Figure 5) indicated that the 100% treatment exerted an inhibitory effect on both shoot and radicle growth. Although intermediate concentrations (75%, 50%, and 25%) showed a tendency toward reduced growth compared with the control, they did not differ statistically from the control, suggesting that the inhibitory effect occurs only at higher concentrations.

**FIGURE 6:**
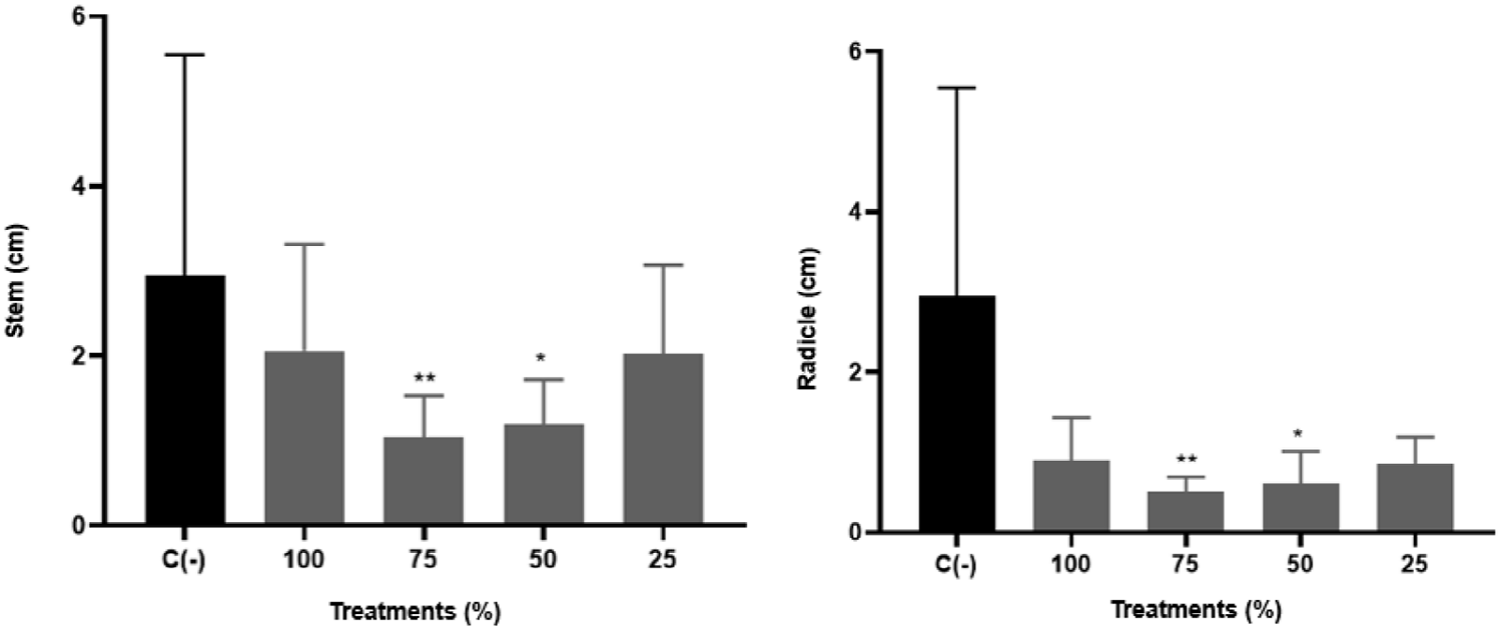
Stem and radicle length (cm) of the EECCMp extract. Values expressed as median and interquartile range (*n* = 25). Differences between groups were assessed using the Kruskal–Wallis test, followed by Dunn's post‐hoc test. ****p < 0.0001 compared to the negative control.

The results obtained for the EECCMp extract (Figure 6) showed a significant reduction in shoot growth at 75% and 50% concentrations compared with the negative control, whereas no significant differences were observed at the other concentrations. A similar pattern was observed for radicle growth, with significant inhibition at 75% and 50%, indicating a more pronounced inhibitory effect at these intermediate concentrations.

In contrast, the 100% and 25% treatments did not differ significantly from the control, suggesting a concentration‐dependent effect of the extract. Greater interference with radicle growth was observed at intermediate concentrations, whereas lower and higher concentrations did not promote significant changes compared with the negative control.

**FIGURE 7:**
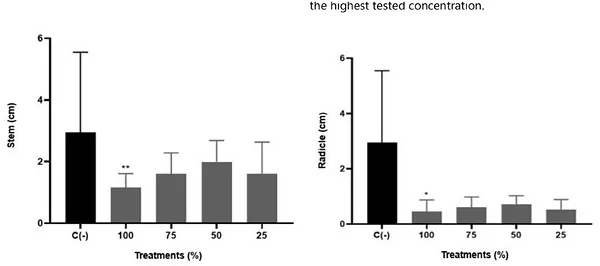
Stem and radicle length (cm) of the EECFMp extract. Values expressed as median and interquartile range (*n* = 25). Differences between groups were assessed using the Kruskal–Wallis test, followed by Dunn's post‐hoc test. ****p < 0.0001 compared to the negative control.

The EECFMp extract (Figure 7) reduced shoot growth at the 100% concentration. At the other evaluated concentrations (75%, 50%, and 25%), no significant differences were observed compared with the control. These findings indicate that the EECFMp extract exerts an inhibitory effect on shoot growth only at the highest tested concentration, whereas intermediate and lower concentrations do not significantly affect this parameter relative to the negative control.

Regarding radicle growth, the effects of the EECFMp extract were observed only at the 100% concentration, at which a significant reduction in growth was detected compared with the negative control. The remaining concentrations (75%, 50%, and 25%) did not differ significantly from the control, although the 25% concentration showed a tendency toward reduced growth. These results indicate that the EECFMp extract exerts an inhibitory effect on radicle growth only at the highest tested concentration.

The allelopathic potential of plant extracts varies as a function of the concentration of bioactive compounds used in germination assays [17]. The exposure of *E. sonchifolia* seeds to *M. pubescens* extracts suggests that the secondary metabolites present may trigger different physiological alterations in the developing plant system. Among the potential effects are the accumulation of reactive oxygen species (ROS), changes in hormonal balance, suppression of energy metabolism, and structural cellular damage, including cell lysis. These processes may partially or completely impair cell division and elongation mechanisms, ultimately resulting in the inhibition of germination [24, 25].

## 4 Conclusion

Based on the data obtained in this study, it was observed that *M. pubescens* exerted negative effects on the germination of *E. sonchifolia* seeds, indicating that the allelochemicals present in this species are capable of adversely affecting seed germination when applied at higher concentrations. Conversely, positive allelopathic effects were observed at lower extract concentrations, suggesting a potential stimulatory effect on seed germination.

Among the analyzed extracts, the leaf extract (EEFMp) at a concentration of 100% demonstrated the greatest potential for weed control, as negative effects were observed across all evaluated parameters under this treatment. Although positive effects were detected at low concentrations for all extracts, the stem bark extract (EECCMp) at a concentration of 50% was particularly promising, promoting increased germination of the tested seeds.

Regarding the chemical composition, the presence of phenolic compounds was particularly noteworthy, especially caffeic acid, which was detected in all three analyzed samples and is known to exhibit a wide range of biologically and economically relevant activities, including allelopathic effects. Further studies investigating the allelopathic potential of this species are needed to explore both applications identified in this research: its use as a bioherbicide and as a biostimulant for the germination of economically and agroforestry‐important species.

Additionally, further investigation of the dose–response relationship observed in the evaluated samples is required to establish reference concentrations for both applications.

## Author Contributions

Conceptualization: M.A.P.S., J.G.M.C., F.F.G.R. and R.N.P.T. Methodology: J.J.F.V., M.E.M.S., J.C.C., F.A.G.S., M.A.P.S. and R.N.P.T. Software: J.B.N., D.O.D.L., J.C.C., F.A.G.S., M.A.P.S. and J.G.M.C. Data curation: D.O.D.L., M.E.M.S. and M.A.P.S. Investigation: J.J.F.V., M.E.M.S., J.C.C., F.A.G.S., M.A.P.S. and F.F.G.R. Validation: J.J.F.V., J.B.N., D.O.D.L. and R.N.P.T. Formal analysis: J.B.N., D.O.D.L. and F.F.G.R. Supervision: J.G.M.C., F.F.G.R. and R.N.P.T. Project administration: J.G.M.C. and R.N.P.T. Writing – original draft: J.J.F.V. and J.G.M.C. Writing – review & editing: M.A.P.S., J.G.M.C. and F.F.G.R.

## Funding

This research received no external funding.

## Conflicts of Interest

The authors declare no conflicts of interest.

## Data Availability

The data supporting the findings of this study are available from the corresponding author upon reasonable request.
